# Co-storage and release of insulin-like peptide-5, glucagon-like peptide-1 and peptideYY from murine and human colonic enteroendocrine cells

**DOI:** 10.1016/j.molmet.2018.07.011

**Published:** 2018-07-30

**Authors:** Lawrence J. Billing, Christopher A. Smith, Pierre Larraufie, Deborah A. Goldspink, Sam Galvin, Richard G. Kay, Jonathan D. Howe, Ryan Walker, Mihai Pruna, Leslie Glass, Ramona Pais, Fiona M. Gribble, Frank Reimann

**Affiliations:** 1 Institute of Metabolic Sciences and MRC-Metabolic Diseases Unit, University of Cambridge, Cambridge, CB0 0QQ, UK; 2 MRC-Laboratory of Molecular Biology, Cambridge, CB2 0QH, UK

**Keywords:** Insulin-like peptide-5 (INSL5), Glucagon-like peptide-1 (GLP-1), peptideYY (PYY), Enteroendocrine, L-cell, LC-MS, Human colonic cultures, 3D-SIM

## Abstract

**Objective:**

Insulin-like peptide-5 (INSL5) is an orexigenic gut hormone found in a subset of colonic and rectal enteroendocrine L-cells together with the anorexigenic hormones glucagon-like peptide-1 (GLP-1) and peptideYY (PYY). Unlike GLP-1 and PYY, INSL5 levels are elevated by calorie restriction, raising questions about how these hormones respond to different stimuli when they arise from the same cell type. The aim of the current study was to identify whether and how INSL5, GLP-1 and PYY are co-secreted or differentially secreted from colonic L-cells.

**Methods:**

An inducible reporter mouse (Insl5-rtTA) was created to enable selective characterisation of *Insl5-*expressing cells. Expression profiling and Ca^2+^-dynamics were assessed using TET-reporter mice. Secretion of INSL5, PYY, and GLP-1 from murine and human colonic crypt cultures was quantified by tandem mass spectrometry. Vesicular co-localisation of the three hormones was analysed in 3D-SIM images of immunofluorescently-labelled murine colonic primary cultures and tissue sections.

**Results:**

INSL5-producing cells expressed a range of G-protein coupled receptors previously identified in GLP-1 expressing L-cells, including *Ffar1, Gpbar1*, and *Agtr1a*. Pharmacological or physiological agonists for these receptors triggered Ca^2+^ transients in INSL5-producing cells and stimulated INSL5 secretion. INSL5 secretory responses strongly correlated with those of PYY and GLP-1 across a range of stimuli. The majority (>80%) of secretory vesicles co-labelled for INSL5, PYY and GLP-1.

**Conclusions:**

INSL5 is largely co-stored with PYY and GLP-1 and all three hormones are co-secreted when INSL5-positive cells are stimulated. Opposing hormonal profiles observed *in vivo* likely reflect differential stimulation of L-cells in the proximal and distal gut.

## Highlights

• A new TET-inducible INSL5 reporter mouse labels INSL5-producing cells.
• INSL5-cells express GPCRs typical of colonic L-cells and respond to cognate agonists.
• By 3D-SIM INSL5, GLP-1 and PYY are located to the same vesicles in colonic L-cells.
• INSL5, GLP-1 and PYY are co-secreted in response to a broad range of stimuli.

## 1 Introduction

Insulin-like-5 (INSL5) is a peptide hormone found to be highly expressed in enteroendocrine cells also producing GLP-1 and PYY in the colon and rectum of human and mouse [1]. The peptide is structurally related to insulin, and is an agonist of the G-protein coupled receptor (GPCR) known as Relaxin/Insulin Like Family Peptide Receptor-4 (RXFP4) [2]. However, unlike GLP-1 and PYY which are anorexigenic, INSL5 has been shown to be orexigenic in mice, and INSL5 production is increased by calorie restriction, contrasting with plasma GLP-1 and PYY concentrations that are elevated after food ingestion [1], [3]. The opposing actions and regulation of INSL5 compared with GLP-1 and PYY raise questions about the mechanisms underlying the secretion of these different products of the intestinal L-cell that have been difficult to address because of the lack of validated tools for identifying live INSL5-secreting cells and measuring INSL5 levels.

Secretion of GLP-1 and PYY is triggered by a variety of stimuli and signalling pathways. In the upper small intestine, physiological stimuli include products of nutrient digestion, such as long chain fatty acids, amino acids and glucose [4]. It is unusual for large nutrient loads to reach as far as the colon, so whilst large intestinal L-cells seem individually capable of responding to nutritional stimuli if they encounter them, it is more likely that they are physiologically regulated by components found at higher concentrations in the colonic lumen, including bile acids and short chain fatty acids, as well as systemic factors such as angiotensin II (Ang II) and arginine vasopressin (AVP) [4], [5], [6]. *Agtr1a* (the receptor for angiotensin II) is exclusively expressed in colonic L-cells, whereas *Avpr1b*, whilst also expressed in small intestinal L-cells, is enriched in colonic L-cells, implying that L-cells exhibit regional differences in GPCR repertoire [5], [6].

Whether INSL5 secretion is triggered by the same stimuli as GLP-1 and PYY is unknown. The opposing findings that INSL5 is increased by calorie restriction whereas GLP-1 and PYY are increased by food ingestion could be explained at the level of tissue exposure or single cell responsiveness. At the tissue level, it could be hypothesised that post-prandial GLP-1 and PYY are released predominantly from L-cells in the small intestine that do not contain INSL5, whereas calorie restriction targets L-cells in the colon and rectum, increasing INSL5 biosynthesis and/or secretion. If the explanation lies at the single cell level, one could hypothesise that GLP-1/PYY and INSL5 are released from the same individual cells but in response to different stimuli. Dissecting these alternatives requires analysis of INSL5-secreting cells and INSL5 secretion. Although we demonstrated INSL5 mRNA and immuno-staining in colonic L-cells, we have been unable to detect INSL5 peptide in tissue homogenates or cell supernatants using a variety of commercial immuno-assays. We therefore recently developed a liquid chromatography – tandem mass spectrometry (LC-MS/MS) based method capable of detecting INSL5 in tissue homogenates and cell supernatants [7]. The use of LC-MS for peptide analysis also enables the co-measurement of a variety of peptides in the same samples.

The aim of this study was to identify stimuli and pathways underlying INSL5 secretion, and to determine whether the peptide is co-released or differentially released compared with GLP-1 and PYY. Our results suggest that INSL5 is co-packaged with GLP-1 and PYY in secretory vesicles of colonic L-cells, and that all three peptides are co-released in response to a broad range of stimuli.

## 2 Methods

### 2.1 Insl5-rtTA mouse

**Figure 1:**
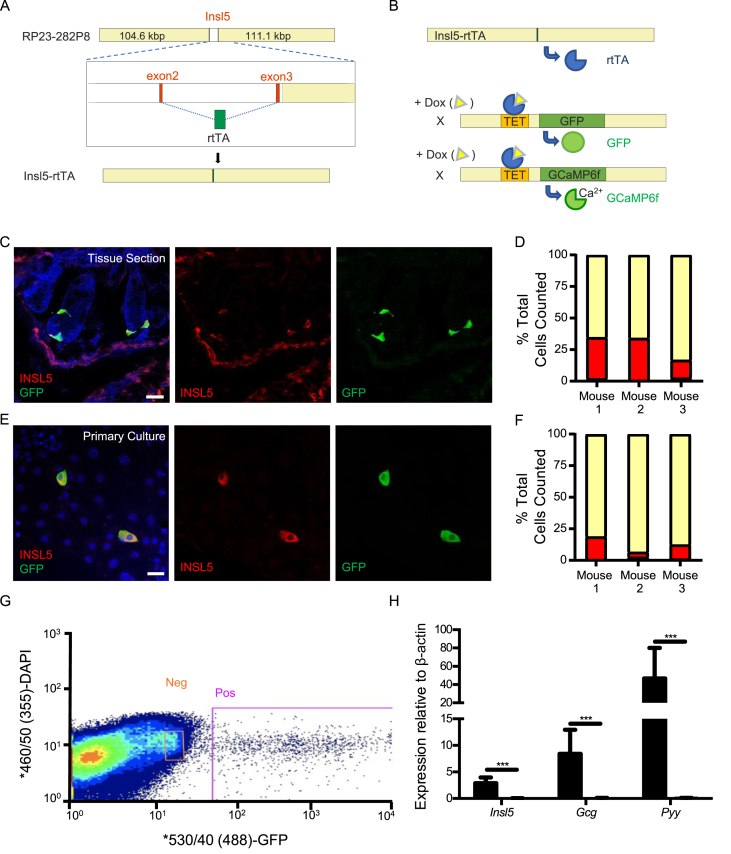
*Insulin-like-5 rtTA mice faithfully report insulin-like-5 producing L-cells.* (A) Scaled diagram of the BAC-transgene used to make Insl5-rtTA mice; Insl5-coding sequence was replaced with the reversed tetracycline activator (rtTA) coding sequence. (B) Cartoon illustrating doxycycline dependent labelling of Insl5-producing cells with either GFP or GCaMP6f. (C&D) Immunofluorescence based assessment of GFP induction in tissue sections (n = 3 mice) following *in vivo* doxycycline induction from Insl5-rtTA/TET-GFP mice. (E&F) Immunofluorescence based assessment of GCaMP6F induction in primary cultures (n = 3 mice) generated from Insl5-rtTA/TET-GCaMP6FΔCMV mice treated *ex vivo* overnight with doxycycline. Bars represent percentage of total cells stained within each of the following subgroups: INSL5 only (red), INSL5 and GFP co-stain (yellow) and GFP only (grey). (G) Representative FACS scatterplot illustrating the subpopulations of cells selected for downstream RT-qPCR. (H) Relative expression of *Insl5, Gcg*, and *Pyy* in FACS GFP+ve and GFP−ve cells following *in vivo* doxycycline induction (n = 6 mice). Bars represent mean 2^ΔCT^ + SEM compared to β-actin. Statistical significance was assessed through ratio paired t-tests applied to 2^ΔCT^. ***p < 0.001.

All animal procedures were approved by the University of Cambridge Animal Welfare and Ethical Review Body and conformed to the Animals (Scientific Procedures) Act 1986 Amendment Regulations (SI 2012/3039). The work was performed under the UK Home Office Project License 70/7824. To create mice with tetracycline-dependent expression of reporter genes in *Insl5*-expressing cells, we replaced the coding region for pro-Insl5 from the start-codon in exon2 (ENSMUSE00000671404) to the Stop in exon3 (ENSMUSE00000796013) in the murine-based BAC RP23-282P8 (Children's Hospital Oakland Research Institute) initially by a counter-selection cassette rpsL-neo (GeneBridges) and subsequently by the rtTA sequence using Red/ET recombination technology (GeneBridges) (Figure 1A) [8]. Briefly, the rpsL-neo {Il5-001/2} or rtTA {Il5-007/8} sequences were amplified by PCR with Phusion-polymerase adding Insl5-gene specific 3′ and 5′ sequences; the sequences of the primers used are given in Suppl.Table S1. Homologous recombination was achieved upon co-transforming the BAC containing Escherichia coli DH10B clone with the PCR product and the plasmid pSC101-BAD-gbaA, which provides the recombination enzymes (GeneBridges). Positive recombinants were isolated using appropriate antibiotic selection and characterized by PCR (primer pairs: Il5-003/4 (1804 bp for rpsL-neo); Il5-004/5 (1048 bp for rtTA); Il5-003/6 (925 bp for rtTA) and restriction analysis (XmaI, AgeI, ApaI and NruI/MluI-digest fingerprints). Identity and correct positioning of the introduced rtTA sequence was confirmed by Sanger sequencing using the oligonucleotides Il5-003, −004, −005 and −006. BAC DNA for microinjection was purified using the large-construct Maxi-Prep kit (Qiagen) and dissolved at ∼1 ng/μL in injection buffer containing 10 mM Tris–HCl (pH 7.5), 0.1 mM EDTA, 100 mM NaCl, 0.03 mM spermine, and 0.07 mM spermidine. Pronuclear injection into ova derived from C57B6/CBA F1 parents and reimplantation of embryos into pseudopregnant females was performed by the Central Biomedical Services at Cambridge University. DNA of pups was isolated from ear clips by proteinase K digestion and screened for the transgene by PCR using the following primer pairs: Il5-003/6, IL5-004/5 and *RM41/42 (β-catenin)*, with the latter serving as a DNA quality control. Initially we received three potential founders, but only two of these passed the transgene to their offspring and faithfully reported Insl5 positive cells when a bidirectional tetracycline reporter (Tre-lacZ/GFP) was crossed in and double-positive offspring were treated with doxycycline in the drinking water (3 mg/ml) (“Insl5-12” and “Insl5-25”) [9]. The transgene copy-number for these two strains was estimated to be ∼1 and ∼5 by quantitative PCR using rtTA and Kcnj11-primer/probes. As no obvious differences were observed in the expression pattern of the two strains, subsequent experiments were performed with Insl5-25, which has been backcrossed for more than eight generations onto a C57B6 background. Mice were housed in individually ventilated cages on a 12-hour light/dark cycle with *ad libitum* access to water and regular chow and were culled by approved schedule 1 methods for tissue collection.

**Figure 2:**
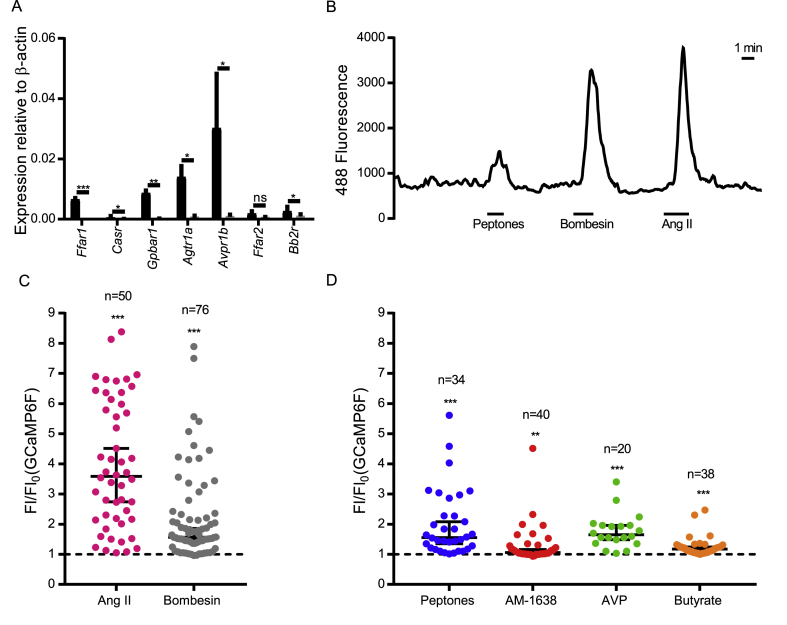
*Activation of Insl5-cell enriched GPCRs triggers Ca*^*2+*^*-responses.* (A) Relative expression of a subset of colonic L-cell GPRCs in FACS GFP+ve and GFP−ve cells following *in vivo* doxycycline induction (n = 3 mice). Bars represent mean 2^ΔCT^ + SEM compared to β-actin. Statistical significance was assessed through ratio paired t-tests applied to 2^ΔCT^. ns = p > 0.05, *p < 0.05, **p < 0.01, ***p < 0.001. (B) Representative trace from a single calcium imaging experiment. (C,D) Intracellular calcium responses of INSL5-producing L-cells to positive controls angiotensin II (10 nM) and bombesin (100 nM) (C) and to agonists of CASR (5 mg/ml peptones), FFA1 (1 μM AM-1638), AVPR1B (10 nM AVP) and FFA2 (2 mM butyrate) (D).

**Figure 3:**
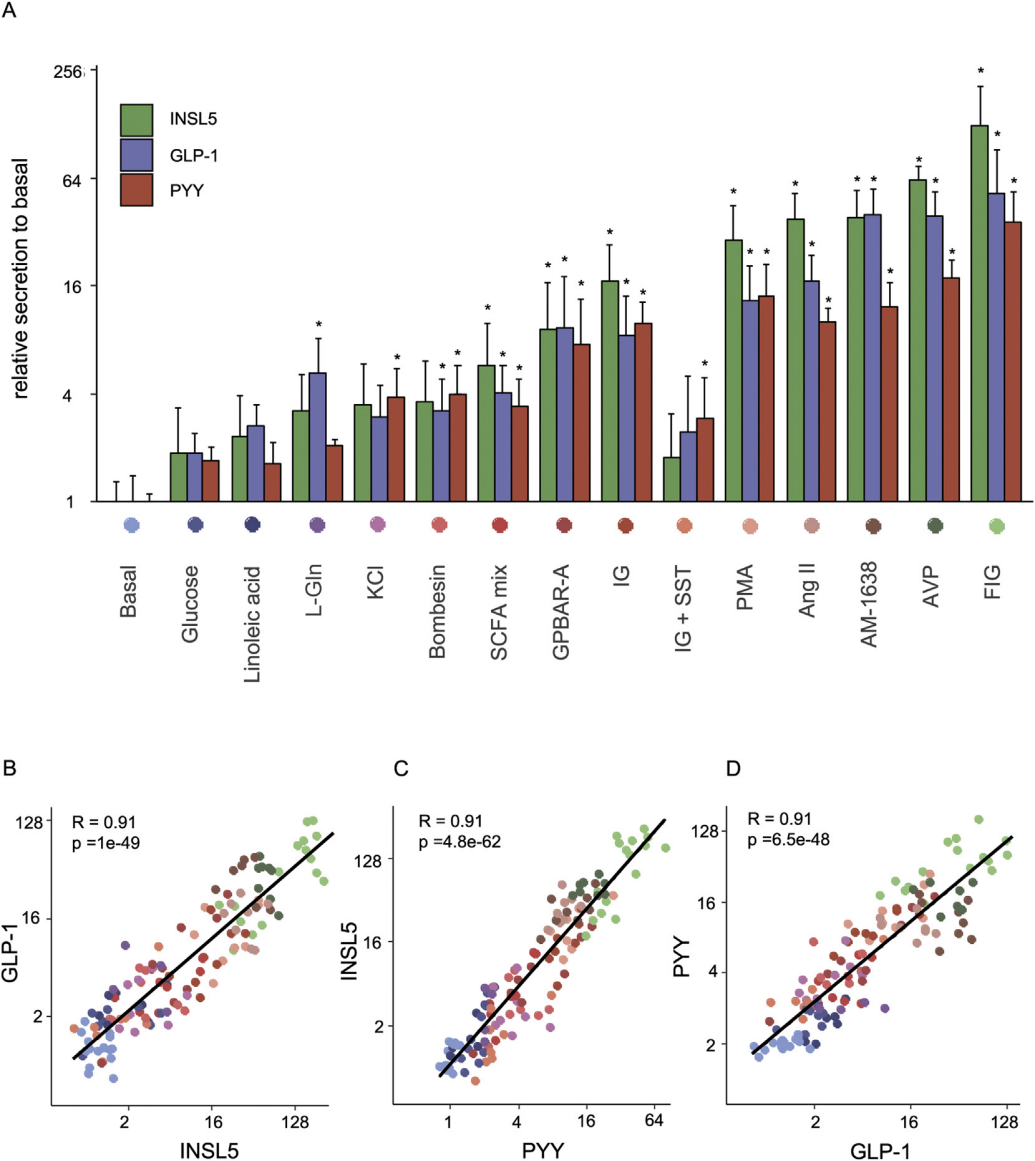
*INSL5, GLP-1, and PYY are co-secreted from murine colonic cultures across a range of stimuli.* (A) Secretion of intact INSL5, GLP-1, and PYY (1–36) from mouse primary colonic cultures treated with different conditions, from at least 3 different cultures treated in duplicates. Data represent mean + SD of the fold increase of peptide quantification (peak area) of the treated condition compared to the mean of the control -treated duplicates of the same culture. *: p < 0.05. (B–D) Correlation plots between GLP-1 and INSL5 (B), INSL5 and PYY (C), and PYY and GLP-1 (D) relative secretion across all different conditions tested. Pearson R correlation coefficient is indicated, and colours are encoded as in (A). Conditions: Glucose (10 mM), Linoleic acid (100 μM), L-glutamine (L-Gln; 10 mM), KCl (70 mM), Bombesin (100 nM), SCFA mix (acetate 3 mM, propionate 1 mM and butyrate 1 mM), GPBAR-A (3 μM), IBMX/glucose (IG; 100 μM/10 mM), PMA (1 μM), Ang-II (10 nM), AM-1638 (1 μM), AVP (10 nM), and forskolin/IBMX/glucose (FIG; 10 μM/10 μM/10 mM); the response to IBMX/glucose was inhibited by SST (IG+SST; 100 nM SST).

**Figure 4:**
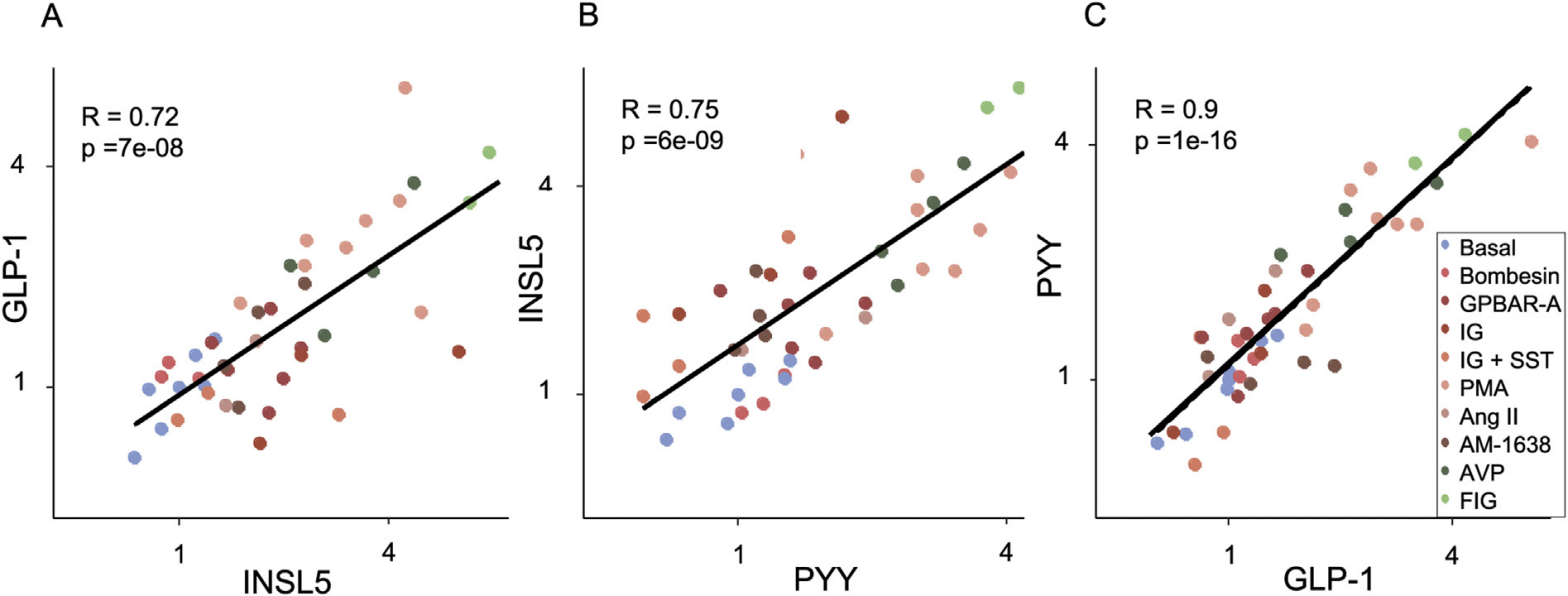
*Co-secretion of INSL5, GLP-1 and PYY from human colonic cultures.* Correlation plots between GLP-1 and INSL5 (A), INSL5 and PYY (B), and PYY and GLP-1 (C) relative secretion from all different conditions as defined in Figure 3 tested on human primary cultures. Pearson R correlation coefficient is indicated.

### 2.2 Isolation of single cells for FACS

Single cell colonic digests were prepared from 3 Insl5-rtTA/TET-GFP and 3 Insl5-rtTA/TET-GCaMP6FΔCMV reporter mice previously orally induced with doxycycline (3 mg/ml) over 5–7 day, using 1 mg/ml collagenase in HBSS (Sigma) as previously described [10]. *Insl5*-expressing cells were isolated through GFP-fluorescence based FACS of single cell suspensions using an Influx Cell Sorter (BD Bioscience, USA; Figure 1G). DAPI-staining, side scatter, forward scatter, and pulse width gates were applied to remove clusters of cells and dead cells. Cells were sorted directly into buffer RLT (Qiagen).

### 2.3 RT-qPCR

RNA was extracted using the RNeasy Micro Plus Kit (Qiagen). Subsequent RT-qPCR was carried out as previously described [11]. The following probes obtained from Applied Biosystems were used: *Insl5:* Mm00442241_m1; *Gcg*: Mm00801714_m1; *Pyy*: Mm00520716_g1; *Ffar1*: Mm00809442_s1; *Casr*: Mm00443375_m1; *Gpbar1*: Mm04212121_s1; *Agtr1a*: Mm01957722_s1, *Avpr1b*: Mm01700416_m1, *Grpr*: Mm01157247_m1 and *Ffar2*: Mm02620654_m1. Relative expression of each gene of interest was calculated by comparison to expression of the housekeeper *β-actin*. The cycle threshold (CT) difference (ΔCT) between each gene and *β-actin* was calculated by subtracting the gene of interest CT from *β-actin* CT. SEM were derived from the ΔCT values with relative gene expression represented in figures as 2^ΔCT^. Ratio paired t-tests were applied to 2^ΔCT^. Undetermined CT values were substituted with a value of 40 (the maximum number of cycles run) to enable statistical analysis.

### 2.4 Murine and human colonic primary culture

All human studies were approved by local ethical review committee (09/H0308/24). Human tissues were obtained from the Human Research Tissue Bank at Addenbrooke's Hospital, Cambridge, UK, and processed within the same day.

Colonic crypts (from colon and rectum for mice samples and from distal colon or rectum for human) were isolated and cultured as previously described [12], [13]. Briefly, tissue was thoroughly washed with cold PBS, outer muscle layers discarded, and samples minced. Crypts were then isolated by incubation in collagenase type XI (Sigma) at 0.4 mg/mL for mouse tissue and 0.5 mg/mL for human tissue and cultured on Matrigel (BD Bioscience) coated plates in high glucose with 10% FBS, 2 mM Glutamine, 100 U/mL penicillin, 0.1 mg/mL streptomycin and 10 μM Y-27632 dihydrochloride (Tocris). Cultures were processed the day after plating after visual inspection for seeded cell density.

### 2.5 Calcium imaging

Colonic crypt cultures were prepared from Insl5-rtTA × TET-GCaMP6FΔCMV mice; the TET-GCaMP6fΔCMV reporter was created by crossing TET-GCaMP6f reporter mice with CMV-Cre mice, which resulted in germ line deletion of the fx-STOP cassette in the original reporter [14]. Expression of the genetically encoded calcium indicator GCaMP6f was achieved *in vitro* by treating the primary cultures with 2 μg/ml doxycycline (Sigma) overnight [15].

Imaging was performed as previously described; briefly GCaMP6F was excited at 488 nm every 2 s with subsequent emission intensity measured through a long pass filter and recorded on MetaFluor [16]. Following background subtraction, intracellular calcium responses to applied stimuli were calculated by dividing the maximum fluorescence measured during drug application by the average maximum recorded 30 s prior to drug application and 30 s following washout. 100 nM bombesin/10 nM angiotensin II were used as positive controls. Cells were excluded from the analysis if they did not respond to either of the positive controls. Significant (p < 0.05) changes in fold intracellular calcium were evaluated using a Wilcoxon ranked sign test by comparing observed fold change in fluorescence to 1 (i.e. no change in fluorescence).

### 2.6 Drug treatments and secretion assays

All reagents were sourced from Sigma except AM-1638, which was a kind gift from Eli Lilly (USA). All drug stocks were made up in water except for AM-1638, which was made up in DMSO. Working solutions were produced by diluting drug stocks in saline buffer containing 4.5 mM KCl, 138 mM NaCl, 4.2 mM NaHCO_3_, 1.2 mM NaH_2_PO_4_, 2.6 mM CaCl_2_, 1.2 mM MgCl_2_, 10 mM HEPES and 1 mM glucose.

For secretion, primary cultures were thoroughly washed with saline buffer for 30 min and then incubated for 1 h at 37 °C with indicated treatments in 600 μL saline buffer with 10 μg/mL BSA. Supernatant was removed and centrifuged for 5 min at 4 °C, 2000 g to remove any cellular debris and immediately snap frozen and stored at −70 °C. Cell cultures were lysed for protein content measurement using a Pierce BCA assay (Thermo Fisher Scientific).

### 2.7 LC-MS/MS

Samples were extracted and concentrated using SPE columns as described [7]. Briefly, 500 μL of supernatant were acidified with 50 μL 1% formic acid, spiked with an internal standard and loaded on primed-μElution SPE plates (Waters) following manufacturer recommendations. Samples were eluted in 2 × 20 μL methanol/acetic acid/water (60%/10%/30%). Mouse samples were analysed intact, whereas human samples were dried then reduced alkylated into 40 μL of 10 mM DTT in 50 mM ammonium bicarbonate for 1 h at 60 °C followed by a 30 min incubation with 10 μL iodoacetamide 100 mM. 50 μL of 0.1% formic acid in water was added to the samples and 40 μL was then injected to the LC-MS. Mouse samples were analysed on a high-flow rate based LC-MS and human samples on a nano flow based LC-MS/MS using a ThermoFisher Ultimate 3000 nano LC system coupled to a Q Exactive Plus Orbitrap mass spectrometer (ThermoScientific, USA) as described, and quantification was performed using XCalibur (ThermoFisher) for amidated GLP-1 and PYY 1–36, intact INSL5 for mouse samples and the N-terminal part of the C-chain of INSL5 (fragment best detected from human INSL5) by measuring peak area for a selected set of *m*/*z* at the retention time corresponding to the peptide of interest [7]. Data were normalised by protein content in the primary lysates and analysed using a Dunn test on relative secretion data or a linear regression calculating Pearson correlation for correlation analysis.

### 2.8 Immunohistochemistry/tissue section

Colonic crypt cultures were cultured overnight as previously described on Matrigel precoated 18 × 18 mm 1.5H glass coverslips (Zeiss) [12], [16]. The following day they were fixed in 4% PFA (Alfa Aesar) for 1 h at room temperature followed by PBS washes. Following brief detergent treatment (0.1% Triton-X 100 in PBS), the cultures were blocked using 10% goat serum in PBS for 15 min followed by GLP-1 (1:100; rabbit; ab22625), PYY (1:500; guinea pig; Progen 16066) and INSL5 (1:1000; rat; Takeda) primary antibodies for 45 min at room temperature. After washing, they were incubated with 1:100 dilutions of the goat secondary antibodies (conjugated with AlexaFluor 488, 555 and 633) for 30 min at room temperature, washed again and finally mounted on glass slides using Hydromount (National Diagnostics). For cell counting, cultures from Insl5-rtTA/TET-GCaMP6FΔCMV mice were treated with 2 μg/ml doxycycline. Following fixation, these cultures were treated with INSL5 (1:2000; rat; Takeda) and GFP (1:1000; rabbit; ab290) primary antibodies followed by goat secondary antibodies (conjugated with AlexaFluor 488 and 647) with an additional 10 min Hoechst nuclear stain (1:2000) treatment.

6–10 μm tissue sections were prepared from 4% PFA fixed whole mouse colons embedded in OCT and sliced using a cryostat onto microscope slides for cell counting and directly onto Poly-Lysine (VWR) coated 18 × 18 mm #1.5H glass coverslips (Zeiss) for 3D-SIM analysis. For cell counting, tissue was prepared from Insl5-rtTA/TET-GFP mice orally induced with 3 mg/ml doxycycline for 5–7 days. Tissue sections were treated similarly to crypt cultures for IHC except for a 1 h block treatment, incubation with primary antibodies overnight at 4 °C and incubation with secondaries for 1 h.

Slides used for cell counting were imaged using an SP8 confocal microscope (Leica Microsystems) with a 20× objective. Cells positive for INSL5 and GFP staining were manually counted from these images and colocalization assessed subsequently.

### 2.9 3D-SIM microscopy

Z-stack images with 0.11 μm spacing were taken for each cell using an ELYRA S.1. microscope (Zeiss) for 3D structured illumination microscopy (3D-SIM) with a pco.edge 5.5 scientific CMOS camera and a 63×/1.4 NA oil immersion objective. These raw images were subsequently reconstructed using ZEN Black (Zeiss) and, for the surfaces, analysis corrected for chromatic aberration by comparison with imaged 100 nm TetraSpeck fluorescent microspheres (ThermoFisher).

#### 2.9.1 Vesicle contents analysis

**Figure 5:**
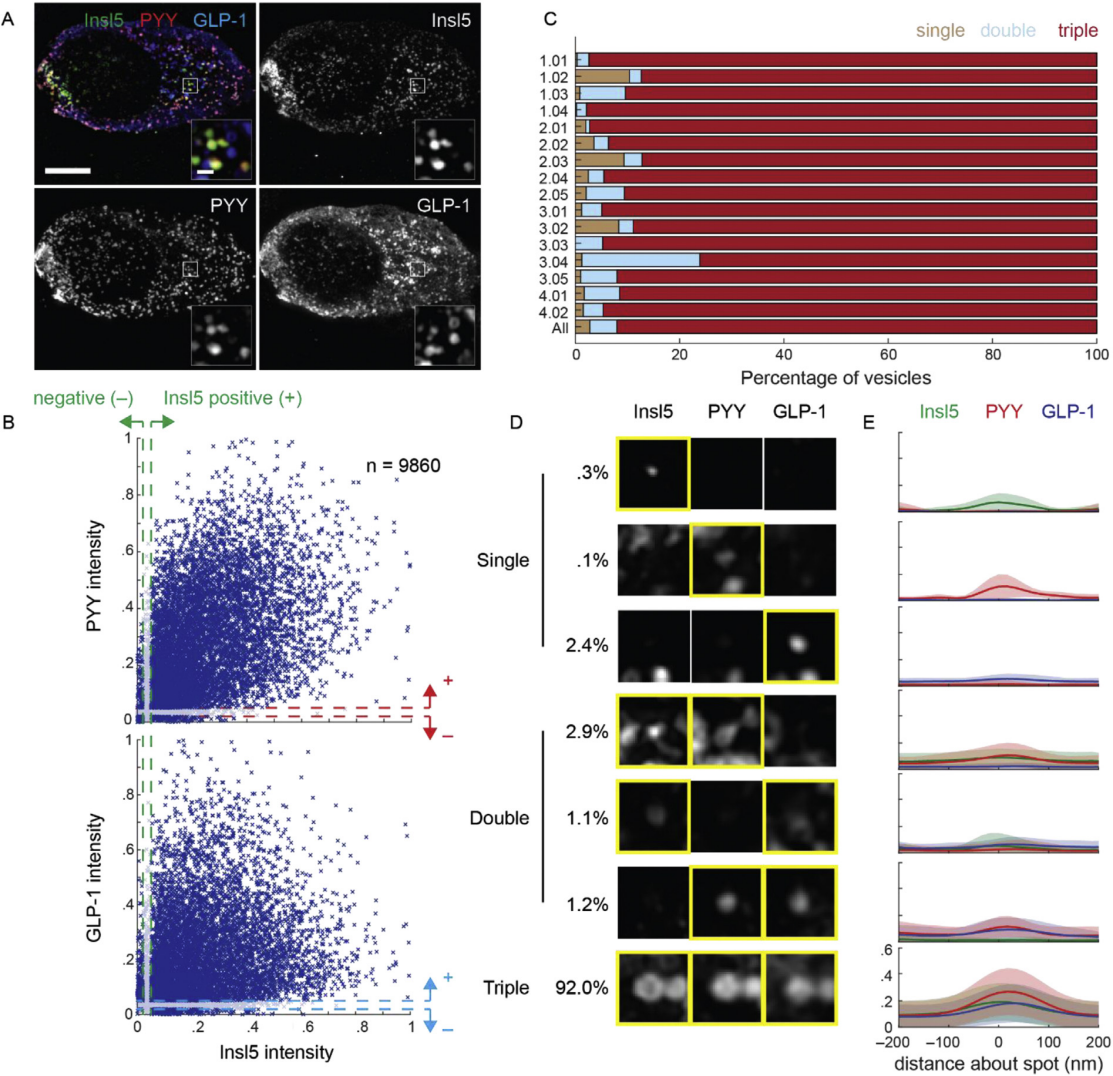
*INSL5, PYY, and GLP-1 are typically localised within the same vesicles in murine colonic cultures.* (A) 3D-SIM image of murine colonic culture labelled for INSL5, PYY, and GLP-1. Images are projected in z. Scale bar = 5 μm. Insets represent individual z-planes of vesicle clusters, scale bar = 500 nm. (B) Scatter of normalised intensity in PYY (top) and GLP-1 (bottom) relative to INSL5 (n = 9860 vesicles, 17 cells, 4 exp.). Dashed lines represent the intensities above (+, 5% of maximum) and below (–, 2%) which vesicles were considered positive or negative, respectively, for each peptide. (C) Percentage of vesicles per cell that contain single peptides (brown), two peptides (blue; double), or all three (red; triple). IDs represent the experiment (*e*) and cell (*c*) numbers, in the format *e.cc*. The final line (‘All’) represents the percentages of vesicles across all experiments. (D) Example vesicle signals in each INSL5, PYY and GLP-1 categorised by whether the vesicle contains only a single peptide (first three rows), pairs of peptides (next three rows), or all three peptides (bottom row). Percentages represent percentage of vesicles within each category. (E) Mean intensity profiles through vesicle signals for each category in D for INSL5 (green), PYY (red) and GLP-1 (blue). Shaded regions represent standard deviation.

**Figure 6:**
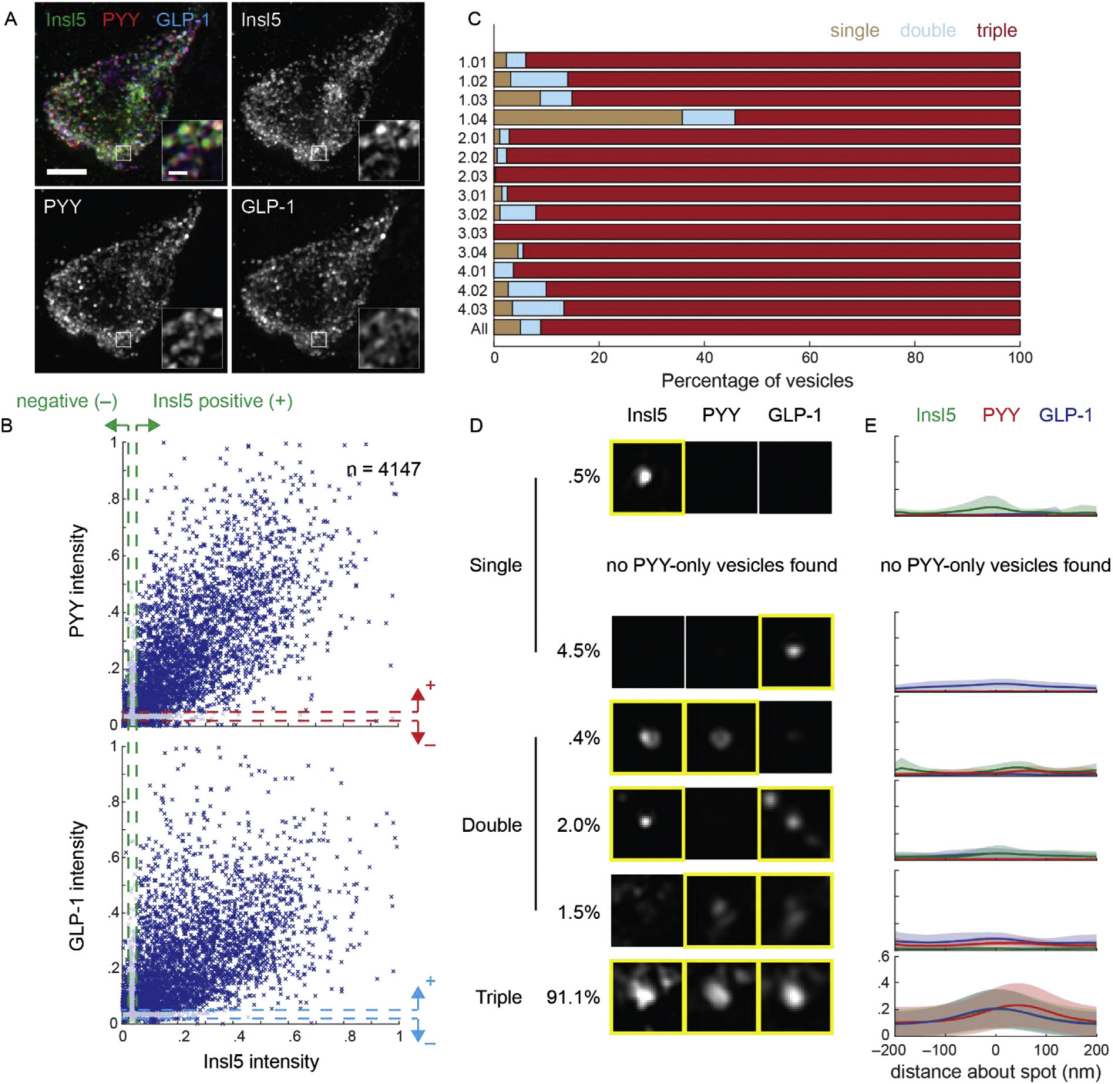
*INSL5, PYY or GLP-1 are typically localised within the same vesicles in murine colonic tissue.* (A) 3D-SIM image of murine colonic tissue labelled for INSL5, PYY, and GLP-1. Images are projected in z. Scale bar = 3 μm. Insets represent individual z-planes of vesicle clusters, scale bar = 500 nm. (B) Scatter of normalised intensity in PYY (top) and GLP-1 (bottom) relative to INSL5 (n = 4147 vesicles, 14 cells, 4 exp.). Dashed lines represent the intensities above (+, 5% of maximum) and below (–, 2%) which vesicles were considered positive or negative, respectively, for each peptide. (C) Percentage of vesicles per cell that contain single peptides (brown), two peptides (blue; double), or all three (red; triple). IDs represent the experiment (*e*) and cell (*c*) numbers, in the format *e.cc*. The final line (‘All’) represents the percentages of vesicles across all experiments. (D) Example vesicle signals in each INSL5, PYY and GLP-1 categorised by whether the vesicle contains only a single peptide (first three rows), pairs of peptides (next three rows), or all three peptides (bottom row). Percentages represent percentage of vesicles within each category. (E) Mean intensity profiles through vesicle signals for each category in D for INSL5 (green), PYY (red) and GLP-1 (blue). Shaded regions represent standard deviation.

Analysis of 3D-SIM images with GLP-1, PYY and INSL5 stained cells was performed using MATLAB (R2017b; MathWorks). Images were exported from ZEN Black (Zeiss) in CZI format, and read into MATLAB using the loci-tools java library (The Open Microscopy Environment). Vesicles were detected by two methods: (1) bright vesicles were detected by splitting the histogram of intensities, and (2) dimmer vesicles were detected by first binarizing the image using Otsu's method thresholding, then selecting only positive regions containing number of pixels, $n_{\text{p}}$, so that 12 $<n_{p}<$ 250. Vesicle detection was performed in all three channels and the positions combined, while removing any duplicates (defined as two coordinates within 2 pixels of one another). A more accurate measurement of vesicle centre position was determined in each channel using mixture-model fitting of 3D Gaussian distributions, resulting in sub-pixel accuracy [17], [18]. The mean 3D position of all vesicles in each of the three channels were made coincident to remove chromatic aberrations. Poorly localised vesicles were removed using pre-filtering by inter-channel separation: it was enforced that for each vesicle the detected position of all three peptide signals were localised <200 nm of one another – any larger distance would suggest a neighbouring vesicle was detected instead. Before intensity measurements, images were first normalised to their maximum intensity pixel, forcing all intensities in the range [0,1]. Background intensity was measured per image per channel as the mean intensity of the full 3D image. Vesicle intensity was measured per channel as the mean pixel intensity within a spherical mask of radius 150 nm centred at the vesicle centre position measured in that channel, minus background intensity. Vesicles were defined as containing a peptide if its intensity is greater than 0.05 (5% of the maximum), and empty if its intensity is smaller than 0.02 (2% of the maximum) (Figure 5, Figure 6). These intensity limits were confirmed using cells labelling GLP-1 simultaneously with the same three secondary antibodies (Fig. S3). The pre-filtering by inter-channel separation was validated by ensuring no bias against single-, double- or triple-labelled vesicles. Greater than 63% of detected vesicles passed this filtering step, with no bias against single-, double-, or triple-labelled vesicles (70%, 67% and 68%, respectively, for murine primary cultures). Intensity line profiles were generated per channel in the microscope's *x*-axis (Fig. S2), centred at the vesicle centre position. Vesicle size was defined per channel as the standard deviation, σ, of the 1D Gaussian distribution fitted to that channel's intensity line profiles (Fig. S2). To avoid erroneous or inaccurate fitting, only fits with R^2^ > 0.75 contributed to distributions of vesicle size. Only the first experiments for murine colonic culture and tissue were used for the analysis shown in Fig. S2. The code used for this analysis is available at https://bitbucket.org/cwissmiff/travis/src.

## 3 Results

### 3.1 TET-system based specific labelling of Insl5-expressing cells

To selectively label *Insl5*-expressing cells, we created a new BAC-transgenic mouse model in which the reverse tetracycline-controlled transactivator (rtTA) is expressed under the control of the *Insl5*-promoter (Figure 1A, B). Insl5-rtTA/TET-GFP and Insl5-rTA/TET-GCaMP6FΔCMV reporter mice were administered doxycycline in drinking water for 5–7 days to induce transgene expression. Tissue sections from induced mice exhibited fluorescent labelling of sporadic cells in the epithelium of the large intestine. By immunostaining of colonic sections from 3 doxycycline-induced mice, 167/232 INSL5-positive cells expressed the fluorescent reporter, whereas only 4/171 reporter-positive cells did not stain for INSL5 indicating that the rate of off-target cell labelling was very low (Figure 1C, D). Primary colonic/rectal cultures from Insl5-rTA/TET-GCaMP6FΔCMV reporter mice were doxycycline-treated ex-vivo overnight, which similarly resulted in a high proportion (205/233) of INSL5-positive cells expressing the fluorescent reporter, with only 3/208 reporter-positive cells having no detectable INSL5 staining (Figure 1E, F). We concluded that the *Insl5* reporter gene in this mouse model could be induced *in vivo* or *ex vivo*, to produce a robust and faithful labelling of INSL5-positive cells.

Fluorescent cells from Insl5-reporter mice and non-fluorescent control cells were purified by fluorescence-assisted cell sorting (FACS), and comprised 0.21 ± 0.05% (n = 6) of the total epithelial cell count (Figure 1G). Insl5-GFP cells were analysed by qPCR to investigate their expression of other known L-cell hormones and nutrient sensing machinery, and exhibited high expression of *Insl5* as well as the gut hormone genes *Gcg* (encoding oxyntomodulin, GLP-1 and GLP-2) and *Pyy* (Figure 1H), all of which were enriched 150–200 times in fluorescent cells compared with non-fluorescent controls.

### 3.2 Nutrient and hormone sensing by Insl5-secreting cells

We used qPCR to measure expression of GPCRs previously implicated in the detection of nutrient and hormonal signals in L-cells (Figure 2A). *Insl5*-labelled cells had enriched expression of *Ffar1*, *Gpbar1*, and *Casr*, involved in the detection of long chain fatty acids, bile acids and amino acids/peptones, respectively [11], [19], [20]. They also had high and enriched expression of *Agtr1a* and *Avpr1b*, previously implicated in the stimulation of GLP-1 and PYY release by angiotensin-II (Ang-II) and AVP, respectively [5], [6].

Insl5rtTA/TET-GCaMP6FΔCMV reporter mice were used to monitor intracellular Ca^2+^ in Insl5-producing cells, following stimulation with agonists of a subset of selected Gq-coupled receptors – Agtr1a, Ffar1, Ffar2, Casr, Grpr, and Avpr1b. Largely consistent with the expression data, Insl5-producing cells exhibited significant Ca^2+^ responses to Ang-II, AM1638 (an FFA1 agonist), butyrate, peptone, bombesin, and AVP, respectively (Figure 2). Interestingly expression of the short chain fatty acid receptor *Ffar2* was relatively low and not significantly enriched in INSL5-producing cells (Figure 2A). However, since butyrate elicited calcium transients in only a small number of the cells tested (Figure 2D), *Ffar2* expression could be restricted to a small subset of INSL5-producing cells and therefore difficult to detect due to sensitivity limitations of RT-qPCR analysis of the total INSL5-producing cell population.

Hormone secretion from primary murine colonic cultures was further investigated by LC-MS, to examine the secretion profiles of GLP-1, PYY, and INSL5 (Figure 3). Cells were stimulated with a variety of secretagogues to examine whether these 3 hormones are released to a similar or different extent under some or all conditions. INSL5 secretion was triggered by these stimuli, with increasing effectiveness in the following order: glucose (10 mM), linoleic acid (100 μM), l-glutamine (10 mM), KCl (70 mM), bombesin (100 nM), SCFA mix (acetate 3 mM, propionate 1 mM and butyrate 1 mM), GPBAR-A (3 μM), IBMX/glucose (100 μM/10 mM), PMA (1 μM), Ang-II (10 nM), AM-1638 (1 μM), AVP (10 nM), and forskolin/IBMX/glucose (10 μM/10 μM/10 mM); the response to IBMX/glucose was inhibited by SST (100 nM). Secretion of GLP-1 and PYY mirrored that of INSL5, as shown by the correlation plots showing that all 3 hormones exhibit similar profiles of responsiveness to strong compared with weak stimuli (R > 0.9, Figure 3B–D)).

Secretion of INSL5, GLP-1, and PYY was also measured by LC-MS in the 4 out of 11 primary cultures of human distal colon in which INSL5 was detectable in cell supernatants (Figure 4). As in the murine cultures we saw a range of response magnitudes following cell incubation with a variety of stimuli, with the greatest responses triggered by a mixture of forskolin, IBMX and glucose. Across the range of stimuli, INSL5, GLP-1, and PYY secretion was largely triggered concomitantly, with high correlations (R > 0.7) between the responses seen for individual hormones.

### 3.3 Vesicular localisation by super-resolution microscopy

As the transcriptomic, Ca^2+^ imaging and secretion data suggested that INSL5, GLP-1, and PYY are co-released in response to the same sets of stimuli, we hypothesised that the 3 peptides would be located in the same vesicles in L-cells. Murine cells in primary culture were triple labelled for INSL5, GLP-1 and PYY, using primary antibodies from different species, and matched secondary fluorescent antibodies of different wavelengths. 3D super-resolution microscopy (3D-SIM) was employed, yielding a 2-fold increase in resolution over that of conventional confocal microscopy (with a 1 Airy Unit pinhole). Using this method, we found that the distribution of deconvolved GLP-1-containing vesicle signals have median 191 ± 6 nm (±95% confidence interval) when labelled with AlexaFluor 488 (Fig. S2). The lower resolving limit of confocal and 3D-SIM microscopes are 180–250 and 100–130 nm, respectively, meaning that almost half of the detected population of vesicles can only be spatially distinguished using 3D-SIM [21], [22].

In the first instance, we examined the degree of co-localisation by mapping the volumes occupied by staining for each hormone in 3-dimensions using the ‘surfaces’ function within Imaris and calculating the proportional overlap between the 3 signals. This method suggested that ∼50% of vesicles stained for only one hormone, 35% for 2 hormones and 15% for all 3 hormones (Fig. S1B). However, we were concerned that the method underestimated % co-localisation and performed a control experiment using a single primary antibody for GLP-1, together with 3 different secondaries with different wavelengths. This experiment should produce a profile of 100% overlap of the 3 colours, but instead it calculated that ∼30% of vesicles were single-labelled, ∼20% double-labelled, and ∼50% triple labelled (Fig. S1H). We concluded that the methodology was flawed and dramatically under-estimated the numbers of double and triple labelled vesicles.

Using this control experiment (anti-GLP-1 targeted by 3 different secondary antibodies), we established an alternative method to analyse individual vesicle overlap. Individual vesicles were first detected by locating the centre of their intensity signal in each channel, before assessing the intensity profiles and maxima. After threshold adjustment, the method reliably detected triple labelling of 95–100% of GLP-1 vesicles using 3 secondary antibodies (Fig. S3). The same parameters were then used to examine primary cultures stained for INSL5, GLP-1, and PYY using the same fluorophores conjugated to the appropriate secondary antibodies. Percent co-localisation was substantially higher than that calculated by the volume overlap method: ∼90% of vesicles were triple-labelled for INSL5, GLP-1 and PYY, ∼5% were double labelled and ∼5% were single labelled (Figure 5C).

A similar method was then applied to tissue sections to examine whether the high percentage of vesicular overlap might have been a consequence of maintaining the cells in primary culture (Figure 6C). Greater variation was seen between the cells in sections compared with those in primary cultures, but in 13 out of 14 cells >80% of vesicles exhibited triple labelling for INSL5, GLP-1 and PYY. Only one cell (out of 14) stood out as different from the others, having ∼40% of vesicles single labelled, ∼10% double labelled and only ∼50% triple labelled.

## 4 Discussion

We describe here a new reporter mouse that enables the identification and purification of murine cells producing INSL5. Transcriptomic analysis revealed co-expression in Insl5-labelled cells of *Gcg* and *Pyy*, together with a range of GPCRs previously identified as being important for the secretion of GLP-1 and/or PYY. By multiplexed LC-MS based measurement of hormone secretion, we found that INSL5 is co-released with GLP-1 and PYY in response to a broad range of stimuli, reflecting our observations using super-resolution microscopy that all 3 hormones are located together in >80% of individual vesicles.

Our Insl5 reporter mouse employs a Tet-on system that could be used to induce transgene expression either *in vivo*, by addition of doxycycline (dox) to the drinking water, or *in vitro* by addition of dox to the culture media. Indeed, gene induction *in vitro* was highly effective, triggering transgene expression in >80% of cells staining positive for INSL5 with very little off-target expression. *In vivo* induction enabled FACS purification of labelled cells for transcriptomic analysis. In FACS-purified cells, we found high expression levels of *Gcg* and *Pyy* together with *Insl5*, consistent with previous identification of INSL5 in colonic cells expressing *Gcg* [1], [23].

Despite the overlap between these hormones at the cell population level, we could not previously ascertain whether Insl5-producing cells might constitute a sub-population L-cells in the large intestine expressing a different repertoire of receptors accounting for the reported responsiveness of INSL5 to calorie restriction as opposed to food ingestion. qPCR analysis of Insl5-labelled cells revealed that they expressed a repertoire of GPCRs similar to that found in *Gcg*-expressing colonic L-cells, including receptors for long chain fatty acids (*Ffar1*), bile acids (*Gpbar1*), amino acids and peptones (*Casr*), Ang-II (*Agtr1a*), AVP (*Avpr1b*), and bombesin (*Grpr*) [5], [6], [11], [19], [20], [24]. Using induced expression of GCaMP6F in Insl5-positive cells, we were also able to demonstrate Ca^2+^ responses triggered by agonists of the subset of these receptors that are Gq-coupled, i.e. AM-1638 (targeting FFA1), peptones, Ang-II and AVP. We additionally observed responses to butyrate, which previous findings on GLP-1 secreting cells would suggest is mediated by free fatty acid receptor 2 (FFA2, also known as GPR43) [25], [26]. Together these findings suggest that *Insl5*-expressing enteroendocrine cells constitute a sub-population of L-cells with markedly similar properties to the remainder of the L-cell population.

There has been considerable debate recently about whether enteroendocrine cells differentially package their hormonal products into vesicles that each contain a single hormone, or whether individual vesicles contain a mixture of hormones [27], [28]. Older literature, for example using immuno-gold electron microscopy, had suggested that in cells producing GLP-1 and PYY, most vesicles contained a mixture of both peptides [29]. Some recent studies, however, have suggested that enteroendocrine hormones are separately packaged into distinct vesicular types, opening the possibility that individual hormones could conceivably be differentially released from a single cell by alternative stimuli [27], [28], [30]. Our data do not support the latter view in the case of the hormones GLP-1, PYY, and INSL5 from the mouse and human gut. This conclusion was based firstly on the secretory responses of primary cultured mouse and human colon to a broad range of stimulus types and intensities which revealed all 3 hormones being released concomitantly, with no stimulus tested favouring secretion of one hormone over another. Further, we used super-resolution microscopy to analyse the proportions of secretory vesicles that were single, double or triple labelled for GLP-1, PYY, and INSL5 in both primary cell cultures and tissue slices and found the majority (>80%) co-stained for all three peptides. We found that the outcomes of these experiments were dependent on the method used for calculating percentage overlap, and that in control experiments, use of a volume-based method over-estimated the proportion of space apparently staining for a single colour fluorescent antibody [27]. We believe this error arose, at least in part, because the method examined the total overlapping volumes of the different coloured secondaries across a whole cell, rather than mapping individual vesicles, and that individual pixels containing a single colour, even if on the edge of a vesicle stained for all 3 colours, counted towards the total proportion of single-labelled vesicles. We avoided this caveat by applying an alternative method that located individual vesicles and scored them positive or negative for each of the three colours.

Whilst these findings do not imply that all enteroendocrine cell types behave similarly, at least for colonic L-cells we conclude that these 3 distinct peptide hormones are not differentially packaged into distinct vesicular pools. We found a greater variability in percentage of triple-labelled vesicles when examining tissue slices rather than primary cultures, and speculate that this is likely to be due to the different ages and environments of the range of cells found *in vivo*. It is likely that cells switch on and off expression of different hormones during their lifespan or in response to conditions in their microenvironment, so cells are likely to contain a mixture of vesicles made at different times when different hormonal promoters were active, reflecting the history of the individual cell.

## 5 Conclusions

Having recently developed a LC-MS/MS based method for the detection of INSL5 in mixed epithelial cultures, which we previously failed to detect with commercially available immuno-assays, we now report that colonic *Insl5*-expressing cells co-secrete INSL5 with PYY and GLP-1 in response to a number of stimuli *in vitro* [1], [7]. Whilst post-prandial plasma levels of GLP-1 and PYY are likely dominated by small intestinal L-cells that are more exposed to nutrients after a meal, what governs colonic L-cell and especially INSL5 secretion physiologically remains elusive. Given the failure of the commercially available immuno-assays to detect INSL5 in mixed epithelial culture supernatants, which we now have proven by LC-MS/MS to be present and secreted in a regulated fashion, INSL5 levels *in vivo* in relation to food intake and/or restriction should be re-investigated when more reliable assays (including LC-MS/MS with improved sensitivity) become available.

Given our findings that GLP-1, PYY, and INSL5 are largely co-stored and released, it seems unlikely that the different plasma dynamics of INSL5, levels of which appear to fall after re-feeding whereas GLP-1/PYY levels rise acutely post-prandially, can be explained by differential regulation of their secretion at the single cell level. A more likely explanation is a dominant effect of small intestinal L-cells in the acute phase, which do not express *Insl5* [1]. Plasma INSL5 levels may drop following re-feeding as a result of unidentified inhibitory factors or by a reduction in stimulation. Longer term, between meals, stimuli in the colon such as bacterial metabolites, together with systemic factors may stimulate INSL5 secretion. A full understanding of INSL5 physiology *in vivo* is, however, still lacking, with different reports suggesting it exhibits orexigenic activity, modulates hepatic glucose production and stimulates insulin secretion [1], [23], [31]. Further studies will be required before we can explain the functional importance of this hormone, and why it should be co-stored and secreted with GLP-1 and PYY from colonic L-cells.

## Author's contribution

The Insl5-rtTA mouse model was generated by FR and initially characterised by LG and RP. Further characterisation of the mouse model, expression analysis and Ca^2+^-imaging was conducted by LJB, with help from DAG, RW and MP. Secretion experiments and MS-analysis were performed by PFL with help from SG and RGK. 3D-SIM experiments were performed and analysed by LJB, JH, DAG and CAS. FMG and FR supervised the experiments and all authors contributed to the writing of the manuscript.
