# Supplementary material for: Co-storage and release of insulin-like peptide-5, glucagon-like peptide-1 and peptideYY from murine and human colonic enteroendocrine cells
Source: Mol Metab. 2018 Jul 30;16:65–75. doi: 10.1016/j.molmet.2018.07.011 (PMC6158034; doi:10.1016/j.molmet.2018.07.011)

## Slide 1
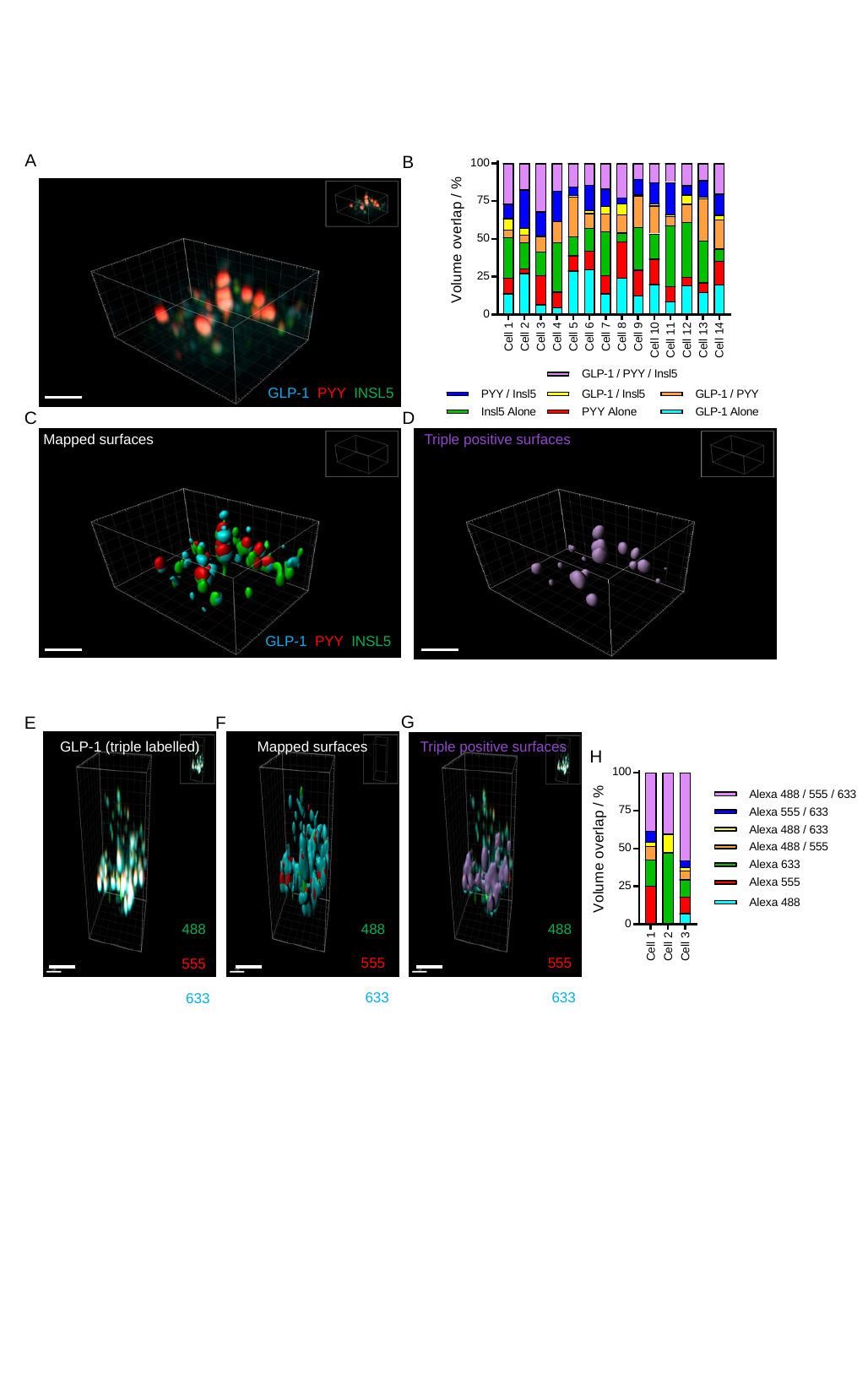

A
B
GLP-1 PYY INSL5
C
D
Mapped surfaces
Triple positive surfaces
GLP-1 PYY INSL5
G
F
E
GLP-1 (triple labelled)
Mapped surfaces
Triple positive surfaces
488
555
633
488
555
633
488
555
633
H

## Slide 2
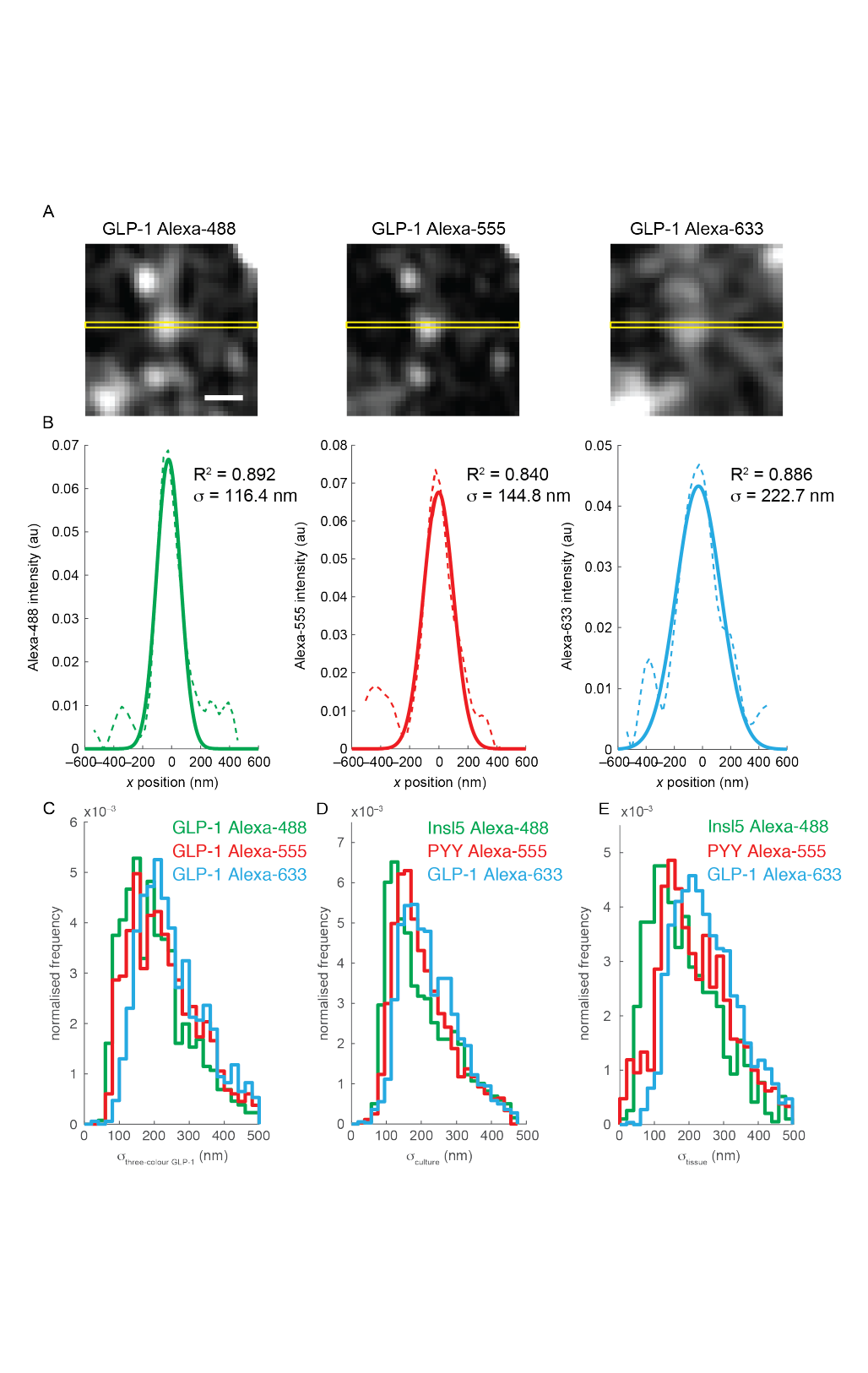

## Slide 3
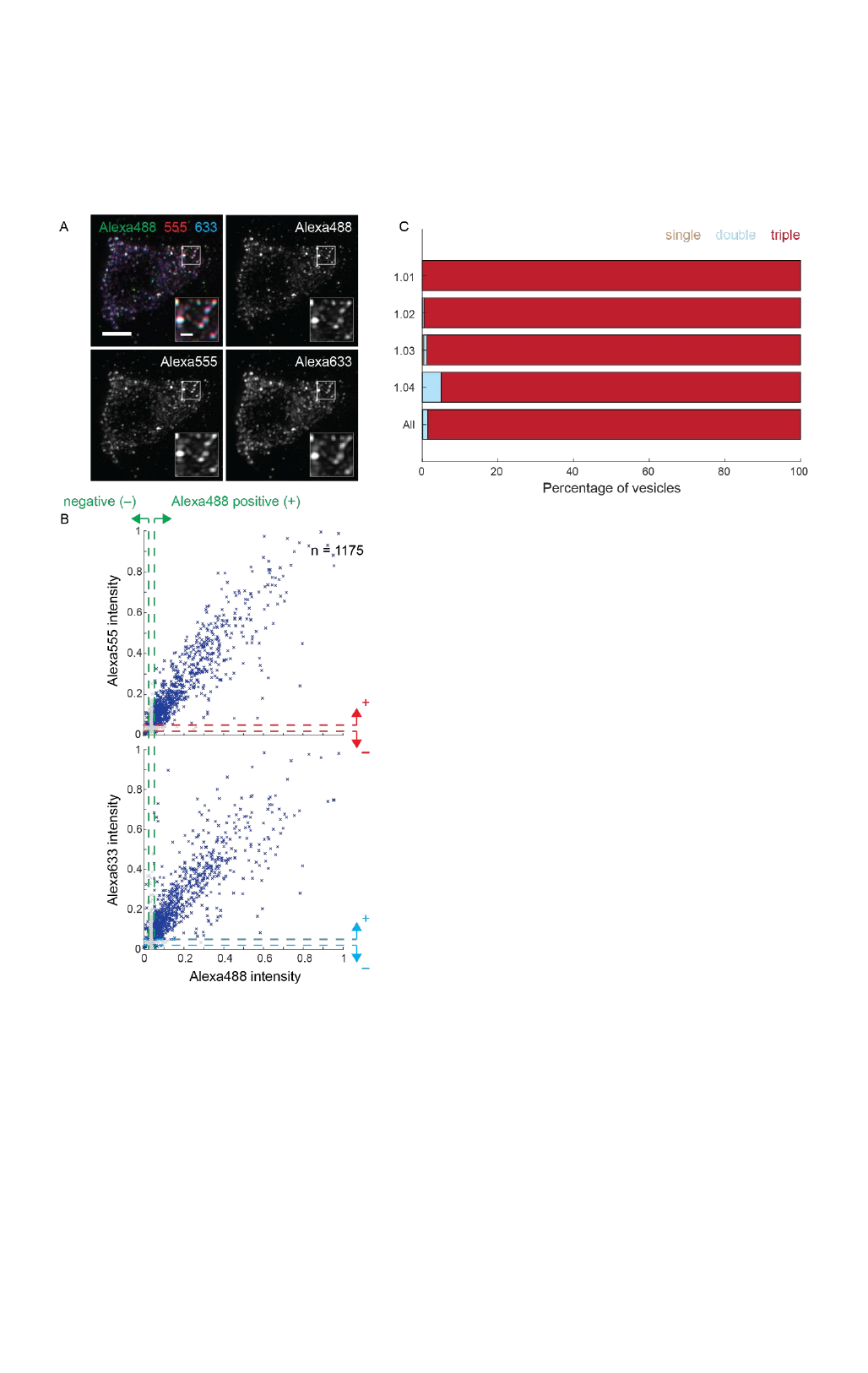

Supplement: Multimedia component 1 [file mmc1.pptx]
