# Supplementary material for: Co-storage and release of insulin-like peptide-5, glucagon-like peptide-1 and peptideYY from murine and human colonic enteroendocrine cells
Source: Mol Metab. 2018 Jul 30;16:65–75. doi: 10.1016/j.molmet.2018.07.011 (PMC6158034; doi:10.1016/j.molmet.2018.07.011)
Supplement: Multimedia component 2 [file mmc2.docx]

**Supplementary material:**

**Supplementary methods:**

*Surfaces Analysis of Secretory Vesicles*

GLP-1, PYY, and INSL5 vesicles within z-stack images of each cell were rendered in 3D by applying the ‘Create Surface’ function on Imaris (Bitplane) to each appropriate channel. Using the ‘Surface-surface Colocalization’ function new surfaces were created from which the degrees of volume overlap between the three types of vesicles were calculated and expressed as a percentage of total volume of GLP-1, PYY, and INSL5 vesicles rendered.

**Supplementary tables and figures:**

Table S1: Primer details

| Name | Sequence |
| --- | --- |
| IL5-001  (-rpsLneo) | AAC TTC AAA ACT CCA CTG GGT ACT GCT GAC CAC ATT GCT TCT CAT TTG CTC TCC GGC AGG ATG GGC CTG GTG ATG ATG GCG GGA TCG |
| IL5-002  (-rpsLneo) | GGA ACT CGA TGG TAG AAC ATT GAG ATG CAT GCT GAA GCC TGC CAA GGG GTT GGG CGC ATC CTA TCA GAA GAA CTC GTC AAG AAG GCG |
| IL-003 | ACA TGG CTG GTC CTC CAC |
| IL-004 | CCC TGA GAC ATC TCT CTA GTC CC |
| IL-005 | ATG TCT AGA CTG GAC AAG AGC AAA GTC |
| IL-006 | TTA CCC GGG GAG CAT GTC AAG GTC |
| IL-007  (-rtTA) | AAC TTC AAA ACT CCA CTG GGT ACT GCT GAC CAC ATT GCT TCT CAT TTG CTC TCC GGC AGG ATG TCT AGA CTG GAC AAG AGC AAA GTC ATA AAC G |
| IL-008  (-rtTA) | GGA ACT CGA TGG TAG AAC ATT GAG ATG CAT GCT GAA GCC TGC CAA GGG GTT GGG CGC ATC TTA CCC GGG GAG CAT GTC AAG GTC |
| RM41 | AAG GTA GAG TGA TGA AAG TTG TT |
| RM41 | CAC CAT GTC CTC TGT CTA TTC |
| rtTA-fw | AAA GTC ATA AAC GGC GCT CT |
| rtTA-rev | ACA GGG TAG GCT GCT CAA CT |
| rtTA-probe | 6-FAM-TGT CGT CAG GCC TTC GAT ACC G-TAMRA |
| mKcnj11-fw | ccc gct tcg tgt cca aga |
| mKcnj11-rev | cag cgt ggt gaa cac atc ct |
| mKcnj11-probe | 6-fam-caa cgt cgc cca caa gaa cat tcg a-BHQ-1 |

*Figure S1: Vesicle overlap assess by volume overlap*

(A) 3D subsection of 3D-SIM imaged primary culture immunofluorescently labelled for GLP-1, PYY and INSL5.

(B) Using the surfaces function on Imaris the degree of volume overlap between stained GLP-1, PYY and INSL5 vesicles was calculated.

(C,D) Examples of mapped surfaces for each individual channel (C) and triple positive vesicles (D).

(E) 3D subsection of 3D-SIM imaged primary culture immunofluorescently labelled for GLP-1 with three secondary antibodies conjugated to different fluorophores.

(F-H) Mapping surfaces of these individual channels (F) together with the computed triple positive surfaces (G) illustrates that the surfaces do not overlap completely (H) as would be expected indicating suboptimal quantification of the degree of overlap between GLP-1, PYY and INSL5 secretory vesicles. Scale bars = 1µm.

Figure S2. *Measuring vesicle size using 1D Gaussian fitting to intensity line profiles.*

(A) 3D-SIM image of a vesicle detected in murine colonic tissue labelled for GLP-1 using Alexa Fluor 488, 555 and 633. Images are projected in $z$ over 5 slices (440 nm). Vesicle intensity line profiles are measured as intensities in the microscope $x$ direction, centred at the vesicle’s detected centre (yellow boxes). Scale bar = 250 nm.

(B) Line profiles of the vesicle shown in A in each Alexa Fluor 488 (green), 555 (red) and 633 (blue) (dashed lines), with fitted 1D Gaussian distributions (full lines). Values given are fitting coefficient, $R^{2}$, and standard deviation of the Gaussian distribution, $\sigma$.

(C) Histogram of standard deviations of fitted 1D Gaussian distributions in murine colonic tissue labelled for GLP-1 using Alexa Fluor 488 (green), 555 (red) and 633 (blue). Mean ($\pm$ std dev) values are $204.6\pm91.9$ nm ($n=655$), $231.9\pm103.6$ nm ($n=679$), and $251.8\pm95.2$ nm ($n=861$), respectively.

(D) Same as C, but for murine colonic culture labelled for INSL5 using Alexa Fluor 488 (green), PYY using Alexa Fluor 555 (red) and GLP-1 using Alexa Fluor 633 (blue). Mean ($\pm$ std dev) values are $198.4\pm91.6$ nm ($n=1676$), $205.1\pm81.9$ nm ($n=1881$), and $224.4\pm80.6$ nm ($n=1896$), respectively.

(E) Same as C, but for murine colonic tissue labelled for INSL5 using Alexa Fluor 488 (green), PYY using Alexa Fluor 555 (red) and GLP-1 using Alexa Fluor 633 (blue). Mean ($\pm$ std dev) values are $187.1\pm97.3$ nm ($n=971$), $220.4\pm107.7$ nm ($n=1074$), and $251.8\pm93.1$ nm ($n=1287$), respectively. Values <100 nm may be the result of fitting Gaussians to experimental artefacts, e.g. hot pixels, higher background intensities in primary tissue.

Figure S3. *Manually setting intensity limits using vesicles labelled with three fluorescent markers.*

(A) 3D-SIM image of murine colonic tissue labelled for GLP-1 using Alexa Fluor 488, 555, and 633. Images are projected in $z$. Scale bar = 3 µm. Insets represent individual $z$-planes of vesicle clusters, scale bar = 500 nm.

(B) Scatter of normalised intensity in Alexa Fluor 555 (top) and 633 (bottom) relative to 488 (n = 1175 vesicles, 4 cells, 1 exp.). Dashed lines represent the intensities above (+, 5% of maximum) and below (–, 2%) which vesicles were considered positive or negative, respectively, for each peptide.

(C) Percentage of vesicles per cell that contain single peptides (brown), two peptides (blue; double), or all three (red; triple). IDs represent the experiment (*e*) and cell (*c*) numbers, in the format *e.cc*. The final line (‘All’) represents the percentages of vesicles across all experiments.
